# Measured vs. Estimated $\dot{V}$O_2max_ in the Yo-Yo Endurance Test: An Exploratory Study in Professional Soccer Players

**DOI:** 10.3390/sports13120424

**Published:** 2025-12-02

**Authors:** Antonio Buglione, Dario Pompa, Marco Beato, Marco Bruno Luigi Rocchi, Cristian Savoia, Maurizio Bertollo, Davide Curzi, Davide Sisti, Fabrizio Perroni

**Affiliations:** 1 Department of Theoretical and Applied Sciences, eCampus University, 22060 Novedrate, Italy; antonio.buglione@uniecampus.it; 2 Behavioral Imaging and Neural Dynamics (BIND) Center, Department of Medicine and Aging Sciences, University “G. d’Annunzio” of Chieti-Pescara, 66100 Chieti, Italy; 3 School of Health and Sports Sciences, University of Suffolk, Ipswich IP4 1QJ, UK; m.beato@uos.ac.uk; 4 Department of Biomolecular Sciences, Section of Exercise and Health Sciences, University of Urbino Carlo Bo, 61029 Urbino, Italy; marco.rocchi@uniurb.it (M.B.L.R.); davide.sisti@uniurb.it (D.S.); fabrizio.perroni@uniurb.it (F.P.); 5 The Research Institute for Sport and Exercise Sciences, The Tom Reilly Building, Liverpool John Moores University, Liverpool L3 5AH, UK; cristiansavoia@gmail.com; 6 Department of Economic, Psychological, Communication, Education and Movement Science, University Niccolò Cusano, 00166 Rome, Italy; davide.curzi@unicusano.it

**Keywords:** aerobic fitness, portable gas analysis, professional soccer, $\dot{V}$O_2max_ estimation, Yo-Yo Endurance Test

## Abstract

Accurate assessment of aerobic fitness is crucial in soccer; however, the validity of field-based predictive tests remains uncertain in professional players. This study examined the relationship between directly measured and estimated maximal oxygen uptake ($\dot{V}$O_2max_) during the Yo-Yo Endurance Test Level 1 (YYET_1_) in professional soccer players and evaluated seasonal changes after six months of training and competition. Seventeen players from an Italian third-division team performed the YYET_1_ in pre- and mid-season conditions, while VO_2max_ was continuously recorded using a portable metabolic system. VO_2max_ was estimated using Bangsbo’s distance-based formula. Linear regression and Bland–Altman analyses were used to assess relationships and agreement between methods. Measured VO_2max_ increased significantly from pre- to mid- season (+13.9%, *p* < 0.001), whereas estimated values showed a smaller rise (+5.2%, *p* < 0.001). The predictive method systematically underestimated VO_2max_ (bias −2.3 to −7.0 mL·kg^−1^·min^−1^), and regression analyses revealed only moderate shared variance (R^2^ = 0.18–0.20) between estimated and measured values. These findings demonstrate that Bangsbo’s equation lacks validity for estimating VO_2max_ in professional players and cannot accurately track aerobic adaptations across a season. For precise physiological evaluation, direct measurement using portable metabolic systems is required, while submaximal soccer-specific protocols may offer practical alternatives for longitudinal monitoring.

## 1. Introduction

Match analysis has shown that professional soccer players typically cover 10–12 km per game, depending on playing position [1,2]. Performance is characterized by the alternation of high- and low-intensity running [3] combined with other demanding movement actions such as jumps, turns, tackles, kicks, and dribbles [4,5]. To fulfill these physical demands, aerobic fitness plays a vital role, with maximal oxygen uptake ($\dot{V}$O_2max_) serving as its key indicator. Specially, $\dot{V}$O_2max_ represents the combined efficiency of central mechanisms—such as cardiac output and oxygen delivery—and peripheral processes like muscle oxygen diffusion and utilization, offering a comprehensive measure of aerobic fitness [6]. Moreover, it is widely recognized as both a determinant of endurance performance and a biomarker of cardiovascular health [6].

Evidence from the Serie A league has shown that high-intensity accelerations are a central determinant of match performance, underscoring the crucial contribution of neuromuscular factors [7]. In parallel, analyses of physiological demands based on the metabolic power approach, which estimates the instantaneous energetic cost of running by combining velocity and acceleration data to derive the overall energy expenditure of performance, have indicated that soccer is predominantly aerobic, with more than 60% of the total energy expenditure sustained by aerobic pathways and roughly 40% derived from anaerobic sources [8]. This ratio highlights the pivotal role of aerobic fitness in sustaining the energetic demands of the game and in enabling rapid recovery from repeated anaerobic efforts [9]. Multiple studies have shown that elite soccer players typically operate at 70–80% of their $\dot{V}$O_2max_ throughout match play [10,11]. Moreover, aerobic fitness has been positively associated with quality of play, positional demands, total distance and high-intensity running covered during match play, as well as with contextual factors and competitive ranking [12,13]. By contrast, only a moderate correlation has been reported with the running-based repeated sprint-ability test (i.e., total sprint time), which typically consists of multiple maximal 20–40 m sprints interspersed with short recovery periods [14].

Additional findings from Serie A showed that the progressive decline in match intensity (i.e., average metabolic power), particularly among midfielders, together with the sustained high-speed demands of forwards and full backs, reinforces the importance of aerobic fitness as a foundation for maintaining high-intensity actions across roles [15]. In line with this, Manzi et al. [16] evaluated professional Serie A players using both maximal ($\dot{V}$O_2max_) and submaximal (velocity at 4 mmol·L^−1^) treadmill tests, demonstrating that these physiological markers were strongly associated with match metrics (e.g., metabolic power) across playing positions. These findings provide robust evidence that aerobic fitness, whether expressed through maximal or submaximal indicators, directly supports players’ ability to cope with the energetic and neuromuscular demands of competition.

In soccer, incremental maximal-load treadmill tests are widely regarded as the gold standard for assessing players’ $\dot{V}$O_2max_. However, their practical application faces several challenges: conducting the test for an entire squad is time-consuming, it requires advanced and costly equipment, and it demands specialized personnel to interpret the results. These factors can significantly limit the feasibility of large-scale evaluations. Consequently, various inexpensive and easy-to-administer field tests have been developed to provide indirect estimates of VO_2max_ in a large number of soccer players [17].

In 1994 [18], Bangsbo adapted the original 20-m multistage shuttle run test into three versions of the Yo-Yo test for soccer players: the Yo-Yo Endurance Test (YYET; levels 1–2), the Yo-Yo Intermittent Endurance Test (YYIET; levels 1–2), and the Yo-Yo Intermittent Recovery Test (YYIRT; levels 1–2) (see Table 1 for an overview of the differences across Yo-Yo test protocols). Given that the running format of Yo-Yo tests more closely mimics the frequent changes in direction (CODs) typical of team sports, whereas continuous linear running is less representative of such demands [19], these protocols have become widely used to assess players’ aerobic fitness [17]. Among them, the YYIET and YYIRT are primarily applied to evaluate the overall performance capacity of soccer players [20,21], whereas the YYET_1_ is predominantly employed to assess aerobic fitness in this population [22]. However, studies have reported varying degrees of correlation between the final level achieved in the YYET_1_ and $\dot{V}$O_2max_: some found very high correlations [23,24], while others observed weaker associations [20]. Notably, the highest correlations (>0.90) were generally reported in active but non-elite individuals rather than in elite soccer players [23,24,25]. More recently, a systematic review and meta-analysis [26] examined the use of different Yo-Yo tests to estimate $\dot{V}$O_2max_ in team sports, including YYET_1_. Although the overall correlation between Yo-Yo performance and $\dot{V}$O_2max_ was large, the two studies specifically focusing on the YYET (level 1 and 2) found no significant association [20,27]. Importantly, both studies involved amateur or youth soccer players, and $\dot{V}$O_2max_ was measured with a treadmill protocol rather than during the Yo-Yo test performance.

Given the current lack of high-quality evidence on the YYET_1_ in professional soccer players, the present study aims to: (1) examine the correlation between directly measured VO_2max_ and the values estimated by Bangsbo’s formula during the execution of the YYET_1_; and (2) evaluate changes in physiological parameters (e.g., VO_2max_, post blood lactate accumulation, _post_BLA) across the pre-season and in-season in a professional soccer team. In this context, our hypothesis was that directly measured $\dot{V}$O_2max_ would show only a moderate shared variance (R^2^) with the values estimated using Bangsbo’s predictive formula [18], which does not represent a valid or sensitive tool for estimating $\dot{V}$O_2max_ or detecting changes in aerobic fitness across the competitive season.

## 2. Materials and Methods

### 2.1. Participants

A sample of 24 professional soccer players from the Italian third division (Serie C; competitive level: Tier 3, according to previous categorization [28]) participated in this study. However, due to turnover in the original sample at the six-months follow up, seven players from the initial group were no longer available and were replaced by seven new players. The final repeated-measures sample comprised 17 players (age: 27.1 ± 3.8 years; height: 178.5 ± 4.3 cm; body mass: 75.2 ± 4.6 kg) who were tested prior to the pre-season (pre-condition) training period (July) as well as six months later, approximately four months into the competitive season, January (post-condition). This study did not perform an a priori power analysis for sample size determination, as it utilized a convenience sample and was designed as an exploratory investigation involving professional soccer players.

Most participants were already familiar with the YYET_1_, as it had been routinely used in the previous seasons by the team’s strength and conditioning coach to assess aerobic fitness. Prior to each testing session, the entire team was familiarized with the equipment and protocols, briefed on the procedures, and provided written informed consent before participating. Ethical approval was granted by the Institutional Review Board of the University Niccolò Cusano (Italy), study protocol number MO3/22 in accordance with the 1964 Helsinki Declaration and its later amendments or comparable ethical standards.

### 2.2. YYET_1_ Assessment Protocol

The YYET_1_ consists of repeated 2 × 20 m shuttle runs (See Table 1) performed between the starting, turning and finishing lines, at a progressively increasing speed, controlled by an audio metronome from a calibrated CD player. Assessment is terminated when participants reach volitional exhaustion or fail on two consecutive occasions to reach the finishing line in time. The distance covered (in meters) was determined by the final stage and shuttle level completed [18]. $\dot{V}$O_2max_ was estimated according to previously established guidelines, using the nomogram proposed by Bangsbo in 1994 for the YYET_1_ [18]:

$$\dot{V}O_{2\max}\;(\mathrm{mL}\cdot {\mathrm{kg}}^{-1}\cdot \min^{-1})=3.5805\times \mathrm{stage}\mathrm{and}\mathrm{step}+9.7696\;(R^{2}=0.9962)$$

Evaluations were performed on a single day after the three-month summer break (off-season phase) and were repeated after six months of training and competitive matches. To minimize circadian rhythms and climate-related factors, testing conditions were standardized: all assessments were conducted at the same time of day (between 9 and 11 a.m. and 3–6 p.m.), on the same natural grass field, approximately two hours after the last meal, and at least 48 h after the last training session. Environmental conditions were consistent with the local seasonal climate, with higher temperatures recorded in July (≈23–32 °C) and milder values in January (≈8–15 °C). Relative humidity showed comparable ranges across testing periods (≈60–70%). To reduce measurement variation, the same experienced investigator (A.B.) conducted all the evaluations.

Prior to the evaluation, soccer players underwent a standardized warm-up lasting 15 min, composed of low-intensity running (40–60% of age-predicted maximal heart rate [HR, e.g., Tanaka et al. [29]]), followed by strolling locomotion and stretching of the lower limb muscles. Finally, during each trial, oxygen uptake (VO_2_) and HR were continuously monitored, and _post_BLA was measured immediately upon test termination.

### 2.3. Oxygen Consumption, Respiratory Exchange Ratio, Heart Rate, and Blood Lactate Accumulation

During the assessment, VO_2_, carbon dioxide production (VCO_2_), ventilation, HR, and _post_BLA were measured, and the respiratory exchange ratio was calculated.

VO_2_ was measured breath by breath through a portable metabolic system (K4b2; Cosmed, Rome, Italy), which has previously shown intraclass correlations coefficients of 0.7–0.9, indicating good reliability [30]. Each player was tested individually in a randomized order. Prior to each trial, the device was calibrated according to the manufacturer’s guidelines: the turbine flowmeter was calibrated using a 3-L syringe and references gases (16% O_2_ and 5% CO_2_). The system’s gas sampling delay (from mouth to analyzer) was determined by synchronizing room-air injections with an auditory cue [19]. During testing, the metabolic system was carried in its dedicated backpack on the participant’s back, with data wirelessly transmitted in real time to a laptop. VO_2_ was recorded continuously throughout each protocol and subsequently averaged over 30 s intervals [19]. HR was monitored with a chest strap transmitter (Polar Team System; Polar Electro Oy, Kempele, Finland). La was assessed at the end of the test and after three minutes of recovery [30] using a portable analyzer (Lactate Pro; Arkray, Tokyo, Japan). For each measurement, a single drop of capillary blood was obtained from the earlobe, and the higher of the two values was retained as the blood lactate accumulation [19,31]. A test was considered maximal when at least three of the following criteria were satisfied: respiratory exchange ratio (RER) ≥ 1.10; peak HR (HR_peak_) ≥ 85% of age-predicted maximal HR (±10 bpm); _post_BLA > 8 mmol, and the presence of a plateau in VO_2_ despite increasing running intensity [20,32].

### 2.4. Statistical Analysis

All data are presented as mean ± standard deviation (SD). Normality of sample data distribution was tested using Shapiro–Wilk test. Moreover, relationships between post-pre (Δ) conditions between $\dot{V}$O_2max_ measured versus estimated were investigated, using linear regression., reporting slopes, intercepts, and coefficients of determination (R^2^). The relationship between distance covered and measured VO_2max_ was not analyzed, as total distance represents a primary component of Bangsbo’s predictive equation and would therefore introduce redundancy due to shared variance. Comparisons between regression parameters (slope and intercept) from the two models were performed using *t*-tests for regression coefficients, while differences between correlation coefficients were evaluated using Fisher’s z-transformation. Effect sizes (ES) were calculated as Cohen’s *d* using the standard deviation of the differences and interpreted according to Cohen’s thresholds (trivial = <0.2, small = 0.2, medium = 0.5, large ≥ 0.8) [33]. This approach allowed assessment of both the strength and consistency of the association between estimation and direct measurement across testing sessions. Agreement between directly measured and estimated $\dot{V}$O_2max_ values was further assessed using Bland–Altman analysis on the same independent datasets (*n* = 17 in pre- and post- condition). For each participant, the mean of the paired measurements (in abscissa) and their difference (estimated − measured $\dot{V}$O_2max_; in ordinate) were reported. The mean difference (bias) was computed with its 95% confidence interval (CI), and the Limits of Agreement (LoA) were defined as bias ± 1.96 × SD of the differences. The 95% CIs of the LoA were obtained according to Bland and Altman [34]. Proportional bias was tested by regressing the differences against the means, with the null hypothesis of a zero-slope assessed using *t*-tests for slope versus expected value. Statistical significance was set at a *p* < 0.05; all analyses were performed using Excel 365 (Microsoft Corporation, Redmond, WA, USA) or RStudio (version 2025.08.0 + 364, R Foundation for Statistical Computing, Vienna, Austria).

## 3. Results

Descriptive statistics for each variable are presented in Table 2.

### 3.1. Regression Comparisons

**Figure 1:**
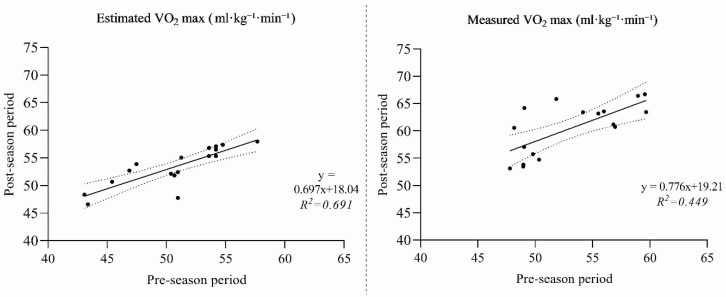
Relationships between pre-season and post-season $\dot{V}$O_2max_ values, both estimated (**left panel**) and measured (**right panel**). Dashed lines represent the 95% confidence interval.

The comparison between the regression coefficients of the estimated and measured $\dot{V}$O_2max_ models did not reveal statistically significant differences. Specifically, the slope coefficients were b = 0.697 and b = 0.776, respectively, with an estimated difference that was not significant (t = 0.82; *p* = 0.42). Likewise, the intercepts of the two models (a = 18.04 vs. a = 19.21) did not differ significantly (t ≈ 0.56; *p* = 0.58). Finally, the variances explained were comparable, with R^2^ = 0.691 for the estimated model and R^2^ = 0.449 for the measured model. Fisher’s z-transformation indicated no significant difference between the two correlation coefficients (z = 0.71; *p* = 0.48). Overall, these results suggest that the two regression models exhibited comparable predictive performance, without statistically meaningful differences in slope, intercept, or explained variance. This indicates that the estimated and directly measured $\dot{V}$O_2_ values share the same degree of variability. However, despite the comparable model behaviour, the absolute values derived from Bangsbo’s predictive formula differed substantially from those measured with the metabolic chart, confirming that the indirect estimation does not reproduce the actual magnitude of $\dot{V}$O_2max_ measured through direct analysis. Furthermore, the regression lines estimating the pre- and post-intervention values (estimated vs. measured) are not significantly different (Figure 1).

### 3.2. Bland–Altman Analysis

**Figure 2:**
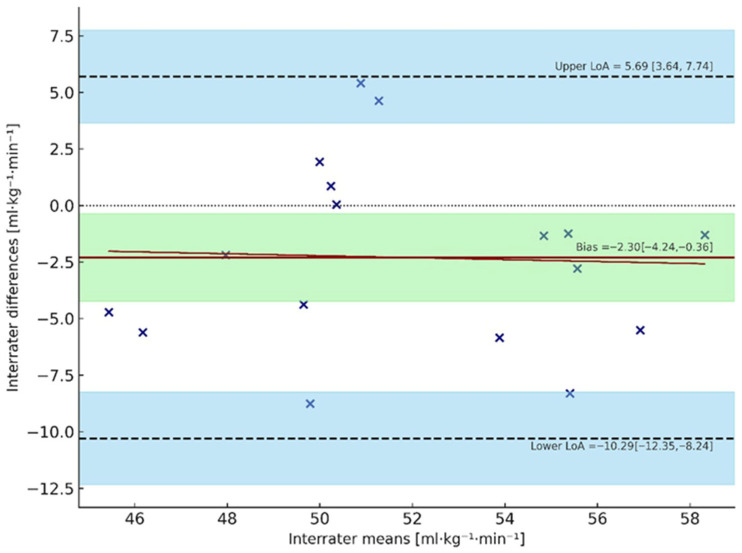
Bland–Altman plot of pre-season $\dot{V}$O_2max_ values. The dashed line represents the line of identity (zero difference), the solid red line represents the mean bias (−2.30 mL·kg^−1^·min^−1^), and the blue shaded dashed lines represents the upper and lower limits of agreement (LoA). The blue shaded zones represent the 95% confidence interval around these LoA, indicating the uncertainty in their estimation. The green shaded area represents the 95% confidence interval of the mean bias. Each cross represents an individual participant, while the red line shows the regression of differences on means, indicating no proportional bias. Values falling outside the LoA suggest poor agreement between measurements.

**Figure 3:**
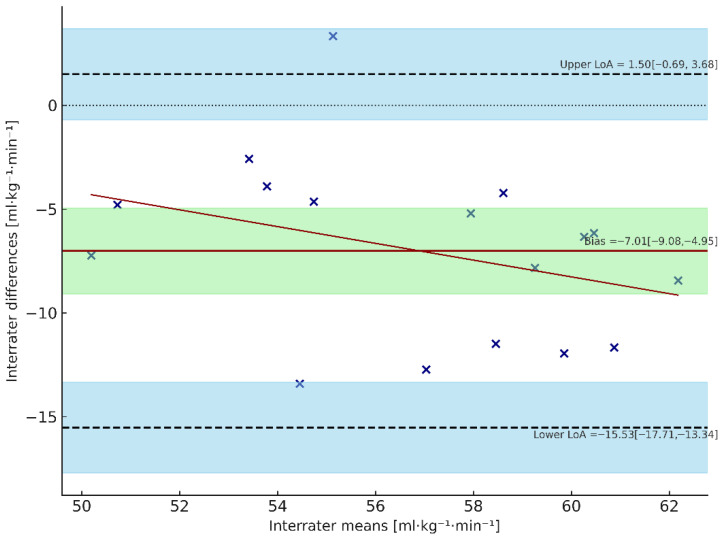
Bland–Altman plot of post-season $\dot{V}$O_2max_ values. The dashed line represents the line of identity, the solid red line represents the mean bias (−7.01 mL·kg^−1^·min^−1^), and the blue shaded dashed lines indicate the upper and lower Limits of Agreement (LoA). The blue shaded areas represent the 95% confidence intervals around these LoA, reflecting the uncertainty in their estimation. The green shaded area represents the 95% confidence interval of the mean bias. Each cross represents an individual participant, while the red line shows the regression of differences on means, indicating no proportional bias. Values falling outside the LoA suggest poor agreement between measurements.

Agreement between estimated and directly measured $\dot{V}$O_2max_ values was evaluated using Bland–Altman method. For pre-measurements (Figure 2), the estimated method slightly underestimated $\dot{V}$O_2max_, with a mean bias of −2.30 mL·kg^−1^·min^−1^ (95% CI: −4.24 to −0.36). LoA ranged from −10.29 to +5.69 mL·kg^−1^·min^−1^, with 95% CIs for the lower and upper LoA of −13.56 to −7.02 and +2.42 to +8.96, respectively. The regression of differences on means showed no evidence of proportional bias (slope test: t = 0.49, *p* = 0.63), indicating that the estimation error was consistent across the range of $\dot{V}$O_2max_ values. For post-measurements (Figure 3), the estimated $\dot{V}$O_2max_ was again lower than measured values, with a mean bias of −7.01 mL·kg^−1^·min^−1^ (95% CI: −9.08 to −4.95). The limits of agreement (LoA) ranged from −15.53 to +1.50 mL·kg^−1^·min^−1^, with 95% CIs for lower and upper LoA of −17.71 to −13.34 and −0.69 to +3.68, respectively. Similarly, no proportional bias was observed (slope test: t = −0.82, *p* = 0.31).

Taken together, these findings indicate a systematic underestimation of $\dot{V}$O_2max_ by the predictive method in both datasets, with a slightly larger bias in the post condition. The dispersion of differences remains comparable between datasets (LoA width ≈ 17 mL·kg^−1^·min^−1^), confirming the same consistent measurement variability.

### 3.3. Seasonal Delta Regression

**Figure 4:**
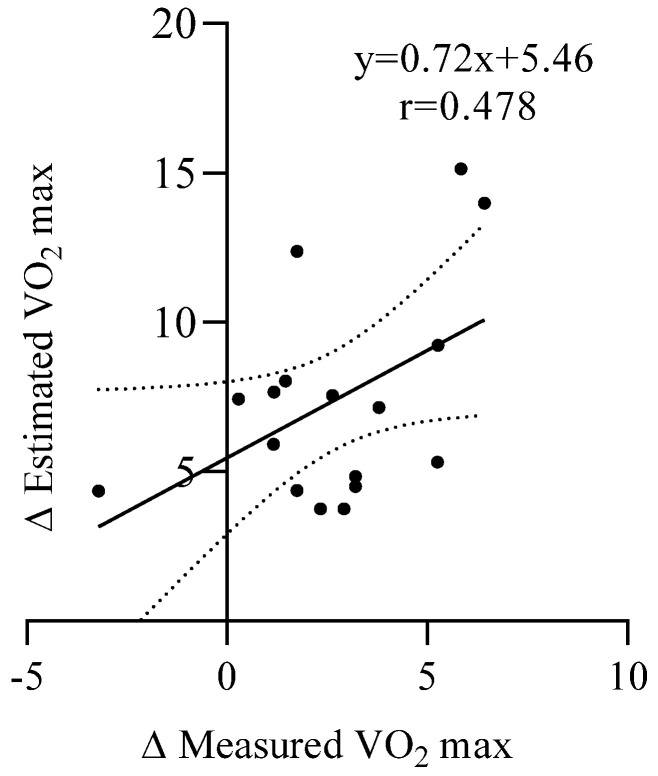
Relationships between post-pre (Δ) between $\dot{V}$O_2max_ measured versus estimated. Dashed lines represent the 95% confidence interval.

A moderate positive relationship was found between the changes in estimated and directly measured $\dot{V}$O_2max_ (Δ $\dot{V}$O_2max_) values across participants (Figure 4). The linear regression analysis yielded the equation y = 0.72x + 5.46, with a correlation coefficient of r = 0.478 (R^2^ = 0.23, *p* = 0.058). This result indicates that individuals who experienced greater improvements in measured $\dot{V}$O_2max_ also tended to show higher increases in estimated $\dot{V}$O_2max_, although the association was slightly above the threshold for statistical significance; this is probably due to the limited sample size.

## 4. Discussion

The primary aim of this study was to examine the correlation between directly measured VO_2max_ and the VO_2max_ estimated from the distance covered in the YYET_1_, evaluated before and after six months of training and official competition in professional soccer players. As hypothesized, this study found a discrepancy between directly measured VO_2max_ and VO_2max_ estimated by Bangsbo’s formula. Specifically, the VO_2max_ estimated from the YYET_1_ was systematically lower than the directly measured values using Bangsbo’s formula [18] (~−4.5% before and ~−13.1% after the six-month period, respectively), highlighting the poor predictive accuracy of the test in our sample of professional soccer players. Secondly, the results showed an increase in the distance covered in the YYET_1_ (+8.8%, *p* < 0.001), alongside an increase in measured VO_2max_ (+13.9%, *p* < 0.001), consistent with previous studies in elite soccer players [17].

Compared with the two studies included in the systematic review and meta-analysis by Tan et al. [26] that examined the association between YYET_1_ and $\dot{V}$O_2max_ in soccer players, our investigation differs in two key aspects: (1) we tested professional soccer players, whereas the two studies using this protocol in the review involved youth and non-professional soccer players [20,27]; and (2) VO_2max_ was measured directly during the YYET_1_, whereas previous research used laboratory treadmill-based protocols as the reference measure. The discrepancy between directly measured VO_2max_ and VO_2max_ estimated by Bangsbo’s formula can be explained, at least in part, by the greater energy cost of shuttle running [35]. Accordingly, the multistage 20-m shuttle run test developed by Leger [36] is calibrated on the energy cost of linear running: it begins at 8 km·h^−1^·and assumes approximately 3.5 mL·kg^−1^·min^−1^ increases every two minutes. Similarly, the YYET_1_ [18] also starts at 8 km·h^−1^·and assigns an initial energy expenditure of ≈27.1 mL·kg^−1^·min^−1^, with 1 MET (3.5 mL·kg^−1^·min^−1^) increments per minute—again based on the energy cost of linear running. In this regard, Savoia et al. [37] reported notable inter-individual differences in the energy cost of submaximal linear running on grass (Cr = 4.66 ± 0.4 J·kg^−1^·m^−1^ at 10.3 km·h^−1^), a coordinatively simpler task than repeated CODs. Such variability suggests that biomechanical and neuromuscular factors can substantially influence running economy even under relatively simple conditions. However, as highlighted by Buglione and di Prampero [38] and, more recently, by Padulo et al. [19], shuttle running involves continuous sequences of accelerations, decelerations, and 180° COD, which non-linearly increase the energy cost compared with straight-line running at the same average speed. This aligns with previous research that examined the energy expenditure associated with shuttle runs performed at varying COD angles (i.e., 180°) [39]. By contrast, the energy cost of linear running is approximately speed-independent at least up to ~20 km·h^−1^ [40]. This higher energy cost of shuttle running is likely one of the main factors causing predictive formulas based solely on distance covered to underestimate VO_2max_ in professional soccer players.

From a neuromuscular perspective, the repeated braking and acceleration phases inherent in shuttle tests impose substantial eccentric demands. In soccer, players typically execute numerous COD across a match, although only a minority involve 180° turns; most CODs occur at smaller angles (<90°) and are more velocity-dominant, whereas sharper angles (≥135–180°) require greater eccentric muscle actions and longer ground contact times [41]. In match play, CODs are typically brief (~0.9 s), with most events occurring at either 0–15° or 105–135°. Moreover, as entry speed increases, the range of attainable COD angles narrows—players tend to execute only small-angle CODs (>5 m·s^−1^)—indicating that movement mechanics are increasingly biased toward braking and re-acceleration demands [42]. Moreover, deceleration ability has been identified as a key determinant of COD performance and is strongly associated with eccentric knee extensor and flexor strength [43]. These neuromuscular requirements contribute to the elevated energy cost of shuttle running, increase inter-individual variability, and may further explain why formulas based on linear running systematically underestimate $\dot{V}$O_2max_. Furthermore, soccer players have been shown to present a higher COD deficit (i.e., the difference between linear sprint and change-of-direction speeds, indicating the athlete’s decreased ability to maintain speed when changing direction) compared with athletes from other team sports, suggesting that COD represents a unique locomotor challenge not fully captured by linear models of energy cost [44]. Applied training studies also demonstrate that adaptations are highly task-specific: COD drills involving sharper cuts (>120°) transfer more broadly across tests, while those with curved or shallow angles show limited transfer [45]. This specificity reinforces the idea that both metabolic and neuromuscular loads must be considered when interpreting performance in shuttle-based field tests.

When comparing our results from the YYET_1_ with studies investigating YYIRT, further insights emerge into these methodological issues. Notably, Krustrup et al. [46] reported moderate-to-strong correlations between YYIRT performance and $\dot{V}$O_2max_ measured in treadmill protocols (r = 0.71) as well as with time to exhaustion (r = 0.79). Although seemingly counterintuitive, as an intermittent shuttle test would be expected to diverge more from treadmill running than a continuous protocol such as the YYET_1_, the discrepancy can be explained by methodological differences. In our study, we directly compared two absolute values—$\dot{V}$O_2max_ measured with a portable metabolic system during the YYET_1_ and $\dot{V}$O_2max_ estimated using a fixed predictive formula [18]. This direct numerical comparison is particularly sensitive to the structural errors of the equation, which systematically underestimated $\dot{V}$O_2max_ (≈−4.5 to −13.1%), thereby inflating bias and reducing explained variance (R^2^ = 0.24–0.34). By contrast, Krustrup and colleagues did not rely on Bangsbo’s formula. Instead, they first established the individual HR–$\dot{V}$O_2_ relationship during a treadmill protocol and then applied it to estimate $\dot{V}$O_2_ during the YYIRT. This individualization minimized variability due to differences in efficiency, energetic cost, and cardiac response. Moreover, their analysis focused on the association between aerobic fitness and functional performance (distance covered, time to exhaustion), rather than on the agreement between two absolute values. This approach naturally produces stronger correlations because it reduces statistical noise and highlights shared variance in aerobic fitness across testing modalities. In this sense, the higher correlations reported for the YYIRT do not necessarily reflect a closer physiological similarity to treadmill running, but rather the methodological advantage of individualized estimation and relational analysis. Stojanović et al. [47] directly measured $\dot{V}$O_2max_ during the YYIRT_1_ in youth basketball players and found that the values estimated from Bangsbo’s formula were systematically lower than those obtained with a portable metabolic system (≈−6.5 mL·kg^−1^·min^−1^), with only moderate correlations (r ≈ 0.65). Similarly, Ventura et al. [48] examined sub-elite soccer referees and showed that Bangsbo’s equation underestimated $\dot{V}$O_2max_ during the YYIRT_1_ by ~15% compared with treadmill measurements, in both men and women. By introducing population- and gender-specific adjusted formulas, they were able to reduce the estimation error and improve agreement. Taken together, these findings across different sports and populations confirm that predictive equations systematically underestimate $\dot{V}$O_2max_ in Yo-Yo tests, reinforcing the need for direct measurement with portable metabolic systems when the goal is accurate physiological assessment.

Beyond predictive accuracy, our physiological responses confirm that the YYET_1_ elicits near-maximal cardiovascular strain and is suitable for deriving HR_peak_. HR_peak_ differed slightly between assessments (191 ± 8 vs. 187 ± 7 bpm; *p* = 0.01), a statistically significant yet small difference, whereas _post_BLA did not (8.11 ± 1.70 vs. 7.95 ± 1.16 mM; *p* = 0.74). These responses support the conclusion that exhaustion was reached on both occasions [32]. Moreover, the HR_peak_ values are comparable to, if not slightly higher than, those typically observed during maximal incremental treadmill tests [10,20,27]. Finally, while our data showed a clear improvement in $\dot{V}$O_2max_ between July and January, Hoppe et al. [49] reported that average metabolic power during pre-season matches was already stable and showed little variability, whereas indicators of high-intensity efforts fluctuated considerably between games. This pattern indicates that match-derived metrics are limited in their ability to detect aerobic adaptations, reinforcing the usefulness of standardized fitness tests for monitoring changes in aerobic fitness.

### Limitations and Future Directions

Despite our efforts to maintain consistent testing conditions across the pre-season and in-season periods, some limitations should be acknowledged. First, all assessments were conducted on a natural grass field, where surface characteristics inevitably varied between July and December (e.g., drier and firmer in summer vs. heavier and softer in winter). Second, although testing was performed at the same time of day, seasonal differences in ambient temperature and humidity could not be fully controlled, potentially influencing players’ performance. Lastly, external environmental factors such as wind and sunlight exposure may also have introduced uncontrolled variability.

Furthermore, an overlooked aspect in the design of this study is the assessment of excess post-exercise oxygen consumption (EPOC). According to Buglione and di Prampero’s approach [38], measuring EPOC during six minutes of rest after exercise would have allowed for the estimation of the anaerobic component, which could then be used to calculate the average energy cost of the YYET_1_ for each participant. This information would have been useful to determine whether the differences observed in $\dot{V}$O_2max_ between subjects were attributable to aerobic fitness and/or to running efficiency. In addition, it would have been useful to compare $\dot{V}$O_2max_ measured during the YYET_1_ with values obtained from a traditional treadmill-based incremental test, to evaluate and directly compare the outcomes derived from the two approaches.

Future research should explore submaximal fitness tests (SMFT) as practical tools for monitoring aerobic fitness in professional soccer. HR responses to standardized submaximal runs are valid, sensitive, non-fatiguing, and repeatable throughout season [50]. Integrating these tests into soccer-specific formats —such as the circuits proposed by Dello Iacono et al. [51] and Savoia et al. [37]—may enhance ecological validity, which is crucial to ensure the applicability of research findings to real-world scenarios [52]. Laboratory evidence suggests that velocity at lactate threshold and running economy better distinguish endurance capacity than $\dot{V}$O_2max_ alone [53], while anaerobic speed/power reserve may offer complementary insights [54]. Compared to the maximal Yo-Yo test, submaximal methods provide a more practical, repeatable, and less disruptive alternative for longitudinal monitoring [55,56].

## 5. Conclusions

This study is the first to directly measure $\dot{V}$O_2max_ during YYET_1_ in professional soccer players. The findings showed significant improvements in both distance covered and measured $\dot{V}$O_2max_ after six months of training and competition. However, $\dot{V}$O_2max_ estimated using Bangsbo’s distance-based predictive equation was systematically lower than directly measured values (~−4.5 to −13.1%), highlighting the poor accuracy of this approach in elite players. The discrepancy can be largely attributed to the greater energy cost of shuttle running, driven by repeated accelerations, decelerations, and CODs, which are not accounted for by linear running-based formulas. Moreover, the substantial neuromuscular demands of braking and re-acceleration further amplify individual variability, reinforcing the inadequacy of generic predictive models. Together with recent evidence from YYIRT studies, our data indicate that distance-derived estimates of $\dot{V}$O_2max_ are not interchangeable with direct measurements. From a practical standpoint, these results underscore that if the aim is to assess $\dot{V}$O_2max_ accurately in professional soccer, the use of portable metabolic systems during field-based protocols is essential. SMFT may provide a complementary solution for longitudinal monitoring, but maximal predictive equations remain unsuitable for precise physiological evaluation in elite soccer contexts.

## Figures and Tables

**Table 1:** Different types of Yo-Yo tests.

	YYET	YYIET	YYIRT
Modalities	Continuous running	5-s active break between each 40-m run	10-s active break between each 40-m run
Level 1	Starting at 8 km·h^−1^	Starting at 8 km·h^−1^	Starting at 10 km·h^−1^
Level 2	Starting at 11.5 km·h^−1^	Starting at 11.5 km·h^−1^	Starting at 13 km·h^−1^

YYET: Yo-Yo Endurance test; YYIET: Yo-Yo Intermittent Endurance test; YYIRT: Yo-Yo Intermittent Recovery test.

**Table 2:** Descriptive statistics.

Measured	Pre-Season (July)	In-Season (January)	Mean Difference	Effect Size (Qualitative Interpretation)
Measured $\dot{V}$O_2max_ (mL·kg^−1^·min^−1^)	53.04 ± 4.35	60.41 ± 4.78 *	+13.90% (CI 5.56–9.18)	2.09 (large)
Estimated $\dot{V}$O_2max_ (mL·kg^−1^·min^−1^)	50.74 ± 4.21	53.40 ± 3.53 *	+5.24% (CI 1.46–3.86)	1.14 (large)
YYET_1_ Distance (m)	2064.71 ± 288.32	2247.06 ± 241.65 *	+8.83% (CI 100.02–264.69)	1.14 (large)
YYET_1_ Speed (km·h^−1^)	13.51 ± 0.65	13.91 ± 0.52 *	2.96% (CI 0.21–0.59)	1.10 (large)
HR_peak_ (bpm)	190.65 ± 8.98	187.53 ± 6.87 *	−1.64% (CI −5.37–−0.89)	−0.71 (medium)
_post_BLA (mmol·L^−1^)	8.11 ± 1.70	7.95 ± 1.16	−1.97% (CI −1.19–−0.86)	−0.08 (trivial)
Ve (L·min^−1^)	138.66 ± 18.21	151.99 ± 10.40 *	9.61% (CI 5.70–20.97)	0.90

* *p* < 0.001 compared to pre-season; CI = 95% Confidence Interval for mean difference; Effect size Cohen’s *d* thresholds: trivial = <0.2, small = 0.2, medium = 0.5, large ≥ 0.8; $\dot{V}$O_2max_: maximal oxygen uptake; YYIET_1_: Yo-Yo Endurance Test level 1; HR_peak_: peak heart rate; _post_BLA: post blood lactate accumulation; Ve: ventilation.

## Data Availability

The data are available from the corresponding authors upon reasonable request. Due to confidentiality agreements with the professional football club involved, the datasets cannot be made publicly available.
